# Problem-Solving and Mental Health Outcomes of Women and Children in the Wake of Intimate Partner Violence

**DOI:** 10.1155/2014/708198

**Published:** 2014-11-11

**Authors:** John Maddoux, Lene Symes, Judith McFarlane, Anne Koci, Heidi Gilroy, Nina Fredland

**Affiliations:** ^1^Department of Research, Texas Woman's University, 304 Administration Drive, P.O. Box 425589, Denton, TX 76204, USA; ^2^College of Nursing, Texas Woman's University, 304 Administration Drive, P.O. Box 425589, Denton, TX 76204, USA

## Abstract

The environmental stress of intimate partner violence is common and often results in mental health problems of depression, anxiety, and PTSD for women and behavioral dysfunctions for their children. Problem-solving skills can serve to mitigate or accentuate the environmental stress of violence and associated impact on mental health. To better understand the relationship between problem-solving skills and mental health of abused women with children, a cross-sectional predictive analysis of 285 abused women who used justice or shelter services was completed. The women were asked about social problem-solving, and mental health symptoms of depression, anxiety, and PTSD as well as behavioral functioning of their children. Higher negative problem-solving scores were associated with significantly (*P* < 0.001) greater odds of having clinically significant levels of PTSD, anxiety, depression, and somatization for the woman and significantly (*P* < 0.001) greater odds of her child having borderline or clinically significant levels of both internalizing and externalizing behaviors. A predominately negative problem-solving approach was strongly associated with poorer outcomes for both mothers and children in the aftermath of the environmental stress of abuse. Interventions addressing problem-solving ability may be beneficial in increasing abused women's abilities to navigate the daily stressors of life following abuse.

## 1. Introduction

On any given day more than 22,000 children are counted as living in a shelter or transitional housing situation due to domestic violence [1]. This statistic does not include those children who remain in the home and whose mothers have not reached out for assistance. In the United States 15.5 million children are residing in families reporting at least one incident of intimate partner violence in the past year and in seven million cases the violence is severe in nature [2]. Children who are raised by mothers who have experienced interpersonal violence and who have related distress manifested by either or both posttraumatic stress disorder (PTSD) and depression symptoms are at risk for behavioral problems [3, 4]. While the environmental stress and trauma of being in a situation where interpersonal abuse occurs adversely influence a child, maternal mental health problems have an ongoing adverse effect on child development even after leaving the abusive relationship [5]. Conversely, mothers who are mentally healthy may have an ongoing positive influence on child development [6]. In other words even under the most trying circumstances, some children are able to overcome hardship and exhibit positive health outcomes. Further the two most important factors for healthy child development reported in the literature are strong connections with at least one caring adult and school [7]. Usually the most influential caring adult is the mother. However, when mothers are incapacitated in some way as a result of an abusive situation, their responsiveness to concerns of their child(ren) may be diminished as well as their involvement within the school community. Thus cognitive functions such as engaging and problem-solving are less than optimal.

Both depression and PTSD symptoms include negative alterations in cognitive function related to problems in concentration, sleep disturbance, and a decreased interest in activities [8]. These symptoms negatively affect the ability to engage in social problem-solving. It may be that impaired social problem-solving related to mental health symptoms in part explains the adverse effects of poor maternal mental health on child development.

Social problem-solving is conceptualized as real-life problem-solving that serves as both a moderator and mediator of the interrelationship between stressful life events and well-being [9, 10]. Social problem-solving is understood to have 2 major parts. One is problem orientation, or an individual's ability, awareness, and appraisal of problems that results in the motivation to engage in problem-solving. The other is problem-solving style, or the processes an individual follows cognitively and behaviorally to understand life problems and resolve or manage them [11]. There is evidence that social problem-solving is related to mental health symptoms. Ranjbar et al. [12] found a significant relationship between students' social problem-solving abilities and mental health symptoms of somatization, anxiety and insomnia, social dysfunction, and depression. Kasckow et al. [13] found an inverse relationship between PTSD symptoms and social problem-solving skills in a community sample of individuals who reported a lifetime traumatic experience on the Structured Clinical Interview of DSM-IV Axis I Disorders. This adds to the evidence that an intervention designed to develop social problem-solving skills is effective in reducing depression symptoms. Problem-solving therapy (PST) promotes effective social problem-solving through training in problem orientation and in 4 major problem-solving skills [10]. Bell and D'Zurilla [14], in a meta-analysis of studies of the use of PST for depression, found evidence that PST is an effective treatment for depression. Our review of the literature did not uncover studies of social problem-solving related to abused women and their children's behavioral outcomes following exposure to partner violence.

### 1.1. Purpose

The purpose of this study is to increase our understanding of the connection between the social problem-solving skills and mental health of abused mothers, who sought help for the abuse through either a District Attorney's (DA) office or a Woman's shelter, and their children's behavioral outcomes. This study is part of a larger seven-year study analyzing the long-term consequences of partner violence on the health and functioning of 300 women and children. For a detailed description of the goals and methods of the primary study see McFarlane et al. [4].

### 1.2. Research Questions

The research questions for this study are as follows.

1. In abused women who were first time help seekers at either a District Attorney's office or a Woman's shelter, can mental health symptoms of PTSD, anxiety, depression, and somatization be predicted from problem-solving ability when controlling current levels of abuse?
2. In abused women who were first time help seekers at either a District Attorney's office or a Woman's shelter, can children's behavioral dysfunction problems be predicted from maternal problem-solving ability when controlling current levels of abuser?

## 2. Methods 

### 2.1. Design

A cross-sectional, naturalistic design was utilized to collect data from women reaching out for support for domestic violence for the first time. This design was utilized to provide us with a unique sample of first time users of support interventions in the wake of violence. To the best of our knowledge, no longitudinal study has been able to follow this unique sample over time.

### 2.2. Setting

Abused women were recruited at five women's shelters and the District Attorney's Office in an urban metropolis in the Southwestern United States with a population exceeding 4 million.

### 2.3. Population and Sample Size Determination

The study sample is composed of English and Spanish speaking women who sought services either at a Woman's shelter or the District Attorney's office for the first time that is participants had never used shelter services or applied for a protection order in the past. Each participant also had at least one child between the ages of 18 months and 15 years who lived with her at least 50% of the time. If the woman had more than one child in that age range the child participant was selected at random, resulting in a mother-child dyad entering the study. Sample size was determined with G-power. Considering 2 independent samples (i.e., sheltered women and protection order applicants), a conservative effect size of 0.40, a power of 0.90, and alpha of 0.05, 135 women were needed in each group. To allow for attrition sample size was set at 150 mother-child dyads in each group, for a total of 300 women and 300 children. This study uses data collected 20 months into the primary study and has a sample size which is 285 women and 285 children, a retention rate of 95%.

### 2.4. Participants

Among the 285 women in this analysis, ages ranged from 18 to 52 (M = 30.65, SD = 7.64). The average length of time they spent in an intimate relationship with the abuser was 86.47 months (SD = 68.97). Over half of the women in this study identified as Hispanic or Spanish (56.7%) and just over one-third of the sample had less than a high school education (33.7%). The field nurses were bilingual and fluent in Spanish and gave the women the option of taking the survey in Spanish or English but most women elected to take the survey in English (72.3%). The children's ages ranged from 1.5 to 16.42 years (M = 6.88, SD = 4.23). All women had experienced physical or sexual abuse by an intimate partner.

### 2.5. Measures

The Social Problem-Solving Inventory-Revised Short (S) (SPSI-R: S) [15] is derived from the longer 70 item Social Problem-solving Inventory. The SPSI-R: S is designed to assess problem-solving for everyday situations. The 25-item tool includes five subscales that measure either adaptive or dysfunctional problem-solving: positive problem orientation, negative problem orientation, rational problem-solving, impulsivity/carelessness style, and avoidance style. An example of a question to measure adaptive problem-solving is as follows. “After carrying out a solution to a problem, I try to evaluate as carefully as possible how much the situation has changed for the better.” An example of dysfunctional problem-solving is as follows. “When my first efforts to solve a problem fail, I get very frustrated.” Higher scores indicate better functioning in problem-solving. Responses to a 5-point scale are summed to arrive at the total score. Possible responses range from 0 (not at all true) to 4 (extremely true). Reliability and validity of the SPSI-R: Short form (S) has been established [9, 16]. The form takes approximately 10 minutes to complete.

The Brief Symptom Inventory-18 [17] is an abbreviated version of the 53-item Brief Symptom Inventory [18], which is a shortened form of the 90-item Symptom Checklist-90-Revised [19]. The BSI-18 has 18 items and is a self-report scale of statements that measure the individual's level of distress over the preceding seven days (5-point Likert-like scale from 0, not at all, to 4, extremely). The 18 statements measure three dimensions: somatization, anxiety, and depression. The Global Severity Index (GSI) of distress is the sum of the three dimensions. Dimension scores for somatization, anxiety, and depression range from 0 to 24. The total Global Severity Index (GSI) score ranges from 0 to 72, with higher scores indicating higher levels of psychological distress [17]. Reported internal consistency estimates are acceptable (0.74 for somatization, 0.79 for anxiety, 0.84 for depression, and 0.89 for the total GSI). Concurrent validity with the SCL-90-R is high, ranging from 0.91 to 0.96 on both dimensions and total scores. Factor analysis on a clinical sample of 1,134 persons yielded a four-factor solution with two of the factors containing exact items belonging to the somatization and depression scales. The other two factors are composed of items belonging to the anxiety scale and considered acceptable [17]. Example items for depression, anxiety, and somatization, respectively, include “Feeling no interest in things,” “Feeling tense or keyed up,” and “Numbness or tingling in parts of your body.” Respondents are asked to endorse how much they were bothered during the past 7 days by each of the 18 items. For this study, coefficient alpha was 0.91 for the Global Severity Index, 0.86 for anxiety, 0.79 for somatization, and 0.85 for depression.

The 7-item symptom scale screens for posttraumatic stress disorder (PTSD) [20] and is a subset of items from the National Institute of Mental Health Diagnostic Interview Schedule for PTSD. The items were empirically derived in the context of an epidemiological study of PTSD in an urban area of the United States. The 7 items selected were those that most efficiently predicted PTSD diagnostic status [20]. The screen consists of 5 avoidance items and 2 hyperarousal items. Items include the following. “Do you avoid being reminded of the abuse by staying away from certain places, people, or activities?” and “After the abuse are you having more trouble than usual falling asleep or staying asleep?” Respondents rate each item as either “yes” or “no” and adding the number of “yes” responses scores the instrument. When the 7-item scale was evaluated for predictive validity in a National Epidemiological Survey, a score of four or more on the 7-item scale identified cases of PTSD with sensitivity of 78%, specificity of 97%, positive predictive value of 75%, and negative predictive value of 98%. The percentage of correctly classified respondents was 96% [21]. For this study, coefficient alpha was 0.70.

The Danger Assessment Scale (DAS) [22] is a 19-item questionnaire with a yes/no response format designed to assist women in determining their potential risk for becoming victim of intimate partner murder. All items refer to risk factors that have been associated with murder in situations involving abuse. Examples of questions include the following. “Has the physical violence increased in severity or frequency?” and “Has the abuser forced the woman to have sex?” Convergent construct validity of the instrument has been supported by correlations in the moderately strong range, with instruments measuring severity and/or frequency of abuse [23]. Validity for differentiating groups is supported by varying means in groups of women with different levels and severity of abuse. For example, the lowest mean scores were in the nonabused sample, and the highest mean scores were in the hospital emergency room group, indicating an injury had taken place through abuse. Samples of abused women from the community had scores in the middle range [23]. Initial reliability of the instrument was 0.71 [22] and ranged from 0.60 to 0.86 in five subsequent studies [23]. Weighted scoring results in four ranges of danger: less than 8 = variable danger; 8–13 = increased danger; 14–17 = severe danger; and 18 or more = extreme danger. For this study coefficient alpha was 0.66.

The Severity of Violence Against Women Scale (SAVAWS) [24] is 47-item instrument designed to measure threats of abuse (19 items) and physical abuse (28 items). Physical abuse items also include 6 items on sexual abuse. Examples of questions about threats are as follows. “How often has (name of abuser) threatened to hurt you?” and “How often has (name of abuser) thrown an object at you?” Examples of questions about physical assault are as follows. “How often has (name of abuser) kicked you?” and “How often has (name of abuser) punched you?” An example of sexual assault is as follows. “How often did the (name of abuser) make you have anal sex against your will?” Nine factors or subscales are included. Each has been demonstrated valid through factor analytic techniques: symbolic violence and mild, moderate, and serious threats (threats of violence dimension) and mild, minor, moderate, serious, and sexual violence (actual violence dimension). For each item, the woman responds using a 4-point scale to indicate how often the behavior occurred (1 = never, 2 = once, 3 = 2-3 times, 4 = 4 or more times). Scores range from 19 to 76 for the threats of abuse and 28 to 112 for physical assault. Initial internal consistency reliability estimates ranged from 0.92 to 0.96 for a sample of 707 college female students and from 0.89 to 0.96 for a scale of 208 community women [24]. Subsequent reliability scores for abused women have ranged from 0.89 to 0.91 for threats of abuse and 0.91 to 0.94 for assault, respectively [25, 26]. For this study, coefficient alpha was 0.95 for the total scale, 0.90 for threats of abuse subscale, 0.93 for physical abuse subscale, and 0.84 for sexual abuse subscale. At baseline, threat scores ranged from 19 to 76 (M = 41.78, SD = 13.32), sexual abuse ranged from 6 to 22 (M = 8.32, SD = 3.64), and physical abuse scores ranged from 21 to 78 (M = 36.52, SD = 13.88).

### 2.6. Analysis

Preliminary analyses were conducted to test the simple/bivariate relationships among variables and to determine if any additional variables needed to be included in the primary analyses. Additionally, prior research identified a link between severity of violence and danger on mental health outcomes [27]. Based on prior findings and preliminary analyses, current levels of danger, physical abuse, and threats of abuse were included as covariates in the primary regression models. Current levels of sexual abuse could not be included as covariates due to an observed bottoming effect, with the vast majority of participants reporting lowest possible score on sexual abuse.

Given the goal of predicting a dichotomous outcome from one or more predictor variables, a series of logistic regression models was used in the preliminary analysis. This method includes testing the overall model for significance. The effect size for the overall model is expressed as Nagelkerke *R* ^2^. The measure of effect size for each individual predictor is expressed as an odds ratio with values greater than one indicating increased odds of the outcome occurring. The effect size for the individual predictor is expressed as an odds ratio and odds ratios greater than one indicate higher odds of the predicted variable occurring.

## 3. Results 

In order to address the first research question, a series of binary logistic regressions were conducted to test for the impact of problem-solving abilities on maternal mental health symptoms. After controlling for current levels of threats, physical abuse, and danger, clinically significant levels of mental distress were predicted from problem-solving scores (Table 1). As shown, all regression models were significant, all *P*s < 0.05, Nagelkerke *R* ^2^ ranging from 0.157 to 0.389. Increased negative problem-solving scores were associated with significantly greater odds of having clinically significant levels of PTSD (1.063), anxiety (1.118), depression (1.094), and somatization (1.038). Higher levels of avoidance style were associated with significantly lower odds of having clinically significant levels of anxiety (0.957) and depression (0.956). Lastly, higher danger scores were associated with significantly greater odds of having clinically significant PTSD scores (1.101). None of the remaining individual predictors were significant.

In order to address the second research question, another series of binary logistic regressions were conducted to test for the impact of maternal problem-solving abilities on child behavioral functioning. After controlling for current levels of maternal threats, physical abuse, and danger, borderline or clinically significant levels of child behavioral functioning subdomains were predicted from maternal problem-solving abilities (Table 2). Both models were significant, *P*s < 0.01. Higher maternal negative problem-solving scores were associated with significantly greater odds of a child having borderline or clinically significant levels of both internalizing (1.043) and externalizing (1.040) behaviors. Higher maternal levels of danger were associated with significantly higher odds of a child having borderline or clinically significant externalizing behavioral problems, odds ratio = 1.084, *P* < 0.05. None of the remaining individual predictors were significant.

### 3.1. Discussion

Results from these analyses did partially support the first hypotheses in that negative problem-solving was significantly associated with greater odds across all mental health measures tested. Additionally, higher levels of an avoidance problem-solving style were associated with increased rates of anxiety as well as depression. However, other facets of problem-solving ability (e.g., positive problem-solving, negative problem-solving, and impulsive/careless problem-solving) were not significantly associated with mental health outcomes. Results also provided partial support for our second hypothesis by linking higher levels of negative problem-solving to increased odds of having a child with clinically significant levels of both internalizing and externalizing behavioral problems. None of the remaining facets of women's problem-solving scores were significantly related to their children's behavioral problems.

Across all the domains of problem-solving measured, having a predominately negative problem-solving approach was most strongly associated with poorer outcomes for both mothers and their children in the aftermath of abuse. Specifically, greater levels of negative problem-solving were associated with higher levels of maternal mental health symptoms including depression, PTSD, anxiety, and somatization. These results are consistent with prior knowledge of the impact of problem-solving abilities on mental health [10, 28]. Furthermore, these results add to prior knowledge by establishing a link from mother's problem-solving functioning to problematic child behaviors. The results of this study provide support for the negative impacts of abuse to mothers on their children's behavior and offer further evidence for the intergenerational impact of violence [5]. Fredland et al. [5], McFarlane et al. [4], Holmes [3], and Kolar and Davey [29] have reported on the negative effects of abuse across generations.

In this study we also found links between avoidance and increased levels of depression and anxiety. Avoidance, through negative reinforcement, often maintains symptoms of depression and anxiety. As an example, when an individual avoids a distressing situation, the uncomfortable emotions often associated with thoughts of doing such a task tend to diminish immediately following an avoidance behavior; however, in the long term, patterns of avoidance tend to decrease social contact with others, increase anxiety related to daily life tasks, and decrease quality of life, which all are characteristic of depression and anxiety. Put another way, avoidance often results in short term gain (e.g., immediate distress goes away) as well as long-term pain (e.g., disconnection from others). Effective problem-solving, by requiring a problem orientation, may diminish avoidance symptoms and their related untoward consequences.

Results from this study failed to find a link between positive problem-solving, rational problem-solving, or impulsive/careless problem-solving on any of the outcomes of interest, including maternal mental health and child behavioral functioning. Conceptually, these findings do make logical sense in that the majority of mental health concerns are characterized by difficulties with logical and positive thoughts processes. For example, irrational thoughts (being the opposite of rational problem-solving) are often present in individuals with depression, anxiety, and/or PTSD; therefore, it is not surprising that higher levels of rational problems solving were not associated with increased odds of having a mental health diagnosis. A similar explanation may also underlie the nonsignificant paths between these problem-solving approaches and child behavioral function. If mothers are modeling appropriate behaviors based on adaptive problem-solving abilities, it would be conceptually fitting that children would in turn practice these types of behaviors, which would be associated with adaptive childhood behaviors. One potential limitation of the current study is the diverse nature of the sample. Although the Social Problem-Solving Inventory has been found to be valid and reliable in North American as well as Spanish populations, there is evidence that some minor differences in the relationship between the problem-solving orientation may be present among cultures [30]. Further analysis by Morera et al. [31] using the Social Problem-Solving Inventory with US Hispanic college students indicated that problem-solving orientation in this group correlated with decision-making skills. Therefore, we believe that, even in the presence of some cultural differences in approach to decision making which may be present, this analysis gives a valid representation of the relationship between mental health and problem-solving in a diverse sample.

Another limitation of the current study is the direction of the relationships. We know that mental health symptoms often contribute to poor problem-solving and thinking. We also know that poor problem-solving and thinking may also contribute to mental health concerns and intrapersonal distress. That being said, we may never fully be able to determine the directional and causal relationship between mental health symptoms and thinking ability. However, regardless of which came first, results from this study clearly demonstrate a link between problem-solving and mental health. We suspect a bidirectional relationship between maternal problem-solving and maternal mental health. Thus, as problem-solving improves or worsens mental health symptoms of PTSD, depression, and anxiety also improve or worsen. This has major implications for interventions, suggesting that an intervention that targets problem-solving may lead to improved maternal mental health. The direction from maternal problem-solving to child behavior outcomes is likely to be one-directional, from mother to child, at least initially. It may be that as child symptoms become chronic or worsen the relationship becomes bidirectional. Regardless of the direction of each of the relationships it seems likely that an intervention targeting maternal problem-solving will have a positive effect on both maternal and child outcomes.

Therefore, results of this study suggest that interventions that address problem-solving ability may be beneficial in increasing women's abilities to navigate the daily stressors of life and, in turn, have a positive impact on levels of maternal mental health symptoms and child behavior outcomes. Examples of specific interventions that may be effective in achieving this goal include problem-solving therapy [10, 14], cognitive processing therapy (CPT) [32], and other cognitive behavioral approaches, which have proved to be effective in reducing mental health symptoms by increasing problem-solving and other cognitive abilities. Furthermore, these treatments can all be offered in group settings and can be provided by numerous healthcare professionals ranging from nurses to counselors to healthcare advocates.

## 4. Conclusions

There are many negative consequences of intimate partner violence that impact the entire family system. Results from this study have further linked difficulties in problem-solving abilities to increased mental health symptoms for mothers as well as a greater likelihood for child behavioral dysfunctions. Importantly, they also suggest directions for interventions with mothers that may have a positive effect on child behavior outcomes.

## Figures and Tables

**Table 1:** Summary of logistic regression predicting clinically significant mental health diagnosis.

	PTSD^1^	Anxiety^2^	Depression^3^	Somatization^4^
	Odds ratio	Odds ratio	Odds ratio	Odds ratio
Positive PS	0.982	1.006	0.971	0.977
Negative PS	1.063^**^	1.118^**^	1.094^**^	1.038^*^
Rational PS	1.014	0.989	1.004	1.011
Impulsive/careless	0.995	0.976	1.001	1.003
Avoidance style	0.985	0.957^*^	0.956^*^	0.995
Threats	1.037	1.035	1.048	0.991
Physical abuse	0.952	0.949	1.024	1.037
Danger	1.101^*^	1.099	1.028	1.074

Note: ^*^ *P* < 0.05; ^**^ *P* < 0.001; ^1^Model summary: *χ* ^2^(8) = 65.82, *P* < 0.001, Nagelkerke *R* ^2^ = 0.293; ^2^Model summary: *χ* ^2^(8) = 64.77, *P* < 0.001, Nagelkerke *R* ^2^ = 0.385; ^3^Model summary: *χ* ^2^(8) = 65.50, *P* < 0.001, Nagelkerke *R* ^2^ = 0.389; ^4^Model summary: *χ* ^2^(8) = 18.31, *P* = 0.019, Nagelkerke *R* ^2^ = 0.157.

**Table 2:** Summary of logistic regression predicting clinically significant child behavioral problems.

	Internalization^1^	Externalization^2^
	Odds ratio	Odds ratio
Positive PS	0.992	0.986
Negative PS	1.043^**^	1.040^**^
Rational PS	1.010	1.008
Impulsive/careless	1.001	1.003
Avoidance style	0.983	0.977
Threats	1.046	1.017
Physical abuse	0.945	0.914
Danger	1.076	1.084^*^

Note: ^1^Model summary: *χ* ^2^(8) = 34.08, *P* < 0.001, Nagelkerke *R* ^2^ = 0.166; ^2^Model summary: *χ* ^2^(8) = 27.53, *P* = 0.001, Nagelkerke *R* ^2^ = 0.137.
